# Differential Gene Expression in Longissimus Dorsi Muscle of Hanwoo Steers—New Insight in Genes Involved in Marbling Development at Younger Ages

**DOI:** 10.3390/genes11111381

**Published:** 2020-11-21

**Authors:** Sara de las Heras-Saldana, Ki Yong Chung, Hyounju Kim, Dajeong Lim, Cedric Gondro, Julius H. J. van der Werf

**Affiliations:** 1 School of Environmental and Rural Science, University of New England, Armidale 2351, NSW, Australia; hkim33@myune.edu.au (H.K.); gondroce@msu.edu (C.G.); jvanderw@une.edu.au (J.H.J.v.d.W.); 2 Department of Beef Science, Korea National College of Agriculture and Fisheries, Jeonju 54874, Korea; cky95@korea.kr; 3 Hanwoo Research Institute, National Institute of Animal Science, Pyeongchang 25340, Korea; 4 Animal Genomics & Bioinformatics Division, National Institute of Animal Science, Jeonbuk 55365, Korea; lim.dj@korea.kr; 5 College of Agriculture & Resources, Department of Animal Science, Michigan State University, East Lansing, MI 48824, USA

**Keywords:** gene expression, marbling, protein-protein interaction network, Hanwoo, RNA-seq

## Abstract

The Korean Hanwoo breed possesses a high capacity to accumulate intramuscular fat, which is measured as a marbling score in the beef industry. Unfortunately, the development of marbling is not completely understood and the identification of differentially expressed genes at an early age is required to better understand this trait. In this study, we took muscle samples from 12 Hanwoo steers at the age of 18 and 30 months. From the contrast between age and marbling score, we identified in total 1883 differentially expressed genes (FDR < 0.05 and logarithm fold change ≥ 1.5) with 782 genes up-regulated and 1101 down-regulated. Differences in gene expression were higher between the ages x marbling groups rather than between high and low marbling groups. At 18 months of age, the genes *SLC38A4*, *ABCA10*, *APOL6*, and two novel genes (ENSBTAG00000015330 and ENSBTAG00000046041) were up-regulated in the high marbling group. From the protein–protein interaction network analysis, we identified unique networks when comparing marbling scores between different ages. Nineteen genes (*AGT*, *SERPINE1*, *ADORA1*, *FOS*, *LEP*, *FOXO1*, *FOXO3*, *ADIPOQ*, *ITGA1*, *SDC1*, *SDC4*, *ITGB3*, *ITGB4*, *CXCL10*, *ACTG2*, *MX1*, *EDN1*, *ACTA2*, and *ESPL1*) were identified to have an important role in marbling development. Further analyses are needed to better understand the role of these genes.

## 1. Introduction

The Korean breed Hanwoo has been subjected to intense artificial selection for growth and intramuscular fat (marbling) for decades, impacting genomic regions that include genes associated with adipocyte differentiation in lipid and the immune system [1,2]. The artificial selection in Hanwoo has promoted a higher genetic predisposition to accumulate intramuscular fat compared with other breeds [3]. This increases the palatability [4] and the amount of healthy fatty acids (monounsaturated and unsaturated fatty acids) in its meat, making it highly valued by consumers [5,6,7]. Moreover, it has been suggested that intramuscular adipose tissue has an important role in muscle development regulation and structural integrity in muscle [8].

In the beef industry, marbling refers to the white flecks or streaks of intramuscular fat (IMF) between bundles of muscle fibers [9]. Marbling score (MS) is used as a reference for the national standard in Korea to rank the IMF content in meat, therefore, there is a price incentive to increase the marbling in the meat of Hanwoo cattle. Nutritional strategies used to increase marbling scores can be summarized by the use of grain-based diets [10] and increasing the feeding period up to 32 months [11]. Genetic selection for marbling can be based on progeny test results, but in the last decade, it has become possible to find genetic markers based on single nucleotide polymorphisms (SNPs) to predict breeding values and select cattle at an earlier age considering genomic information [12].

Differences in marbling scores have been studied at the genomic (genetic variants) and transcriptomic (transcribed messenger RNA) levels. Multiple SNPs have been identified where variation in SNP genotype is associated with phenotypic variation in marbling, and such SNPs have been identified in nearly all chromosomes [13,14,15]. The genes *LEP*, *CAPN1*, *CAST*, *ADIPOQ*, *PPARG*, *IGF1*, *FN1*, *SORBS1*, *MYL12B*, *SH3KBP1*, *ACSL1*, *MYLK*, *ABCA1*, *PRKCA*, *RUNX1*, *ME1*, *ASAP1,* and *RB1* contained SNPs associated with marbling [14]. The genes *FABP4*, *SCD*, *PPARϒ*, *TITIN*, *NEBULIN,* and *SCD* were differentially expressed between Hanwoo steers with high and low marbling scores [16]. The induced expression of *FABP4* gene promotes the formation of small lipid droplets on bovine stem cells [17]. In a later study, the genes *ADAMTS4*, *CYP51A,* and *SQLE* were up-regulated in muscle of high marbled cattle [18]. The genes *RXRA*, *PPARϒ*, *PLTP*, *SCD*, *NRIH3*, *CPT2*, *ACADL*, *ACOX2*, and *FABP4* were differentially expressed between Hanwoo cattle with high and low intramuscular fat content [19]. In a study combining data on mRNA and miRNA, a significant effect of the miRNA bta-miR-660, bta-miR-16a, bta-miR-27a3p, and bta-miR-2392 on MS was reported [20]. The same authors also identified 15 genes as a target by miRNA from which *KLF11* and *IGF1* regulated fatty acid synthase and lipid metabolism. In addition, protein–protein interaction networks are widely used to identify important nodes (genes) associated with marbling score using information based on the literature [21], and results from gene expression [22] or GWAS [23]. These studies have focused on determining the differences in gene expression at a late stage of growth in cattle, at around 30 months. This gives an association of the genes that are involved in determining the marbling phenotype in the last stage of growth. However, few studies have looked at molecular markers and gene expression effects for the onset of marbling at younger ages [24]. The use of RNA-seq technology in younger cattle could help to find the genes that are involved in pathways at an earlier stage of growth and development of marbling.

The objective of this study was to find the differentially expressed genes between low and high marbling muscle in 18 and 30 months old Hanwoo steers. In addition, we aimed to identify important genes from protein–protein interaction networks that were characteristic in each marbling group and age.

## 2. Materials and Methods

### 2.1. Experimental Design

All the procedures conducted in this study were approved by the animal ethics committee of Hanwoo Research Institute (RDA) in the Korean NIAS (2018-319). Twelve Hanwoo steers, of 12 months old, from two sires were used in the present study. There were differences in the moment of introducing the concentrate diet. Four animals were fed with a concentrated diet from 6 months until 30 months; five animals were grass-fed with pasture from 6 months to 12 months of age and then finished in a feedlot until 30 mo; finally, three animals were fed with pasture from 6 months to 19 months followed by finishing in a feedlot until 30 months. Each of the two sires had at least one offspring in each treatment. All animals were fed with a concentrated diet during the feedlot period from the specified age until 30 months of age. The ingredient composition for the high concentrate diet is detailed in Supplementary Materials Table S1.

Samples from the Longissimus dorsi (LD) muscle from the steers’ left side close to the 13th rib and 1st lumbar vertebra were taken at 18 months of age by biopsy and after the slaughter at 30 months of age. Briefly, for the biopsy, steers were restrained in a hydraulic squeeze chute, hair was removed from the biopsy site, and a local anesthetic (lidocaine HCl; 20 mg/mL; 8 mL per biopsy site) was administered. The biopsy site was cleaned with 70% ethanol on sterile surgical gauze. A 1 to 2 cm incision was made with a sterile scalpel. Biopsy samples (1.0 g) were collected from the L. dorsi muscle using a sterile Bergstrom biopsy needle (custom made diameter: 5.3 mm) following procedures described previously [25,26]. The sampled muscle was preserved in liquid nitrogen and was then stored at −80 °C.

At the end of the experiment (30 months of age), the animals were humanely slaughtered and phenotypic data on carcass weight (CWT) and marbling score (MS) were recorded. The MS was visually scored between the 13th rib and the 1st lumbar vertebra using the scores from 1 to 9, according to the Korean National standard [27]. We used the MS scores to group the animals, five steers were grouped in Low MS (from 1 to 5), while seven steers were grouped as High MS (from 6 to 9). The average CWT at slaughter for the Low and High MS groups were 413.8 kg (SD = 72.4) and 440.3 kg (SD = 47.26), respectively.

### 2.2. RNA Extraction and Library Preparation

Total RNA was extracted from Longissimus dorsi muscle using the TRIzol reagent (Invitrogen, Carlsbad, CA, USA) following the manufacturer’s instructions. Total RNA was treated with DNase1 (0.1%; DNA-Free, Ambion, CA, USA), while the quality and quantity of RNA were assessed using automated capillary gel electrophoresis on a Bioanalyzer 2100 with RNA 6000 Nano Labchips (Agilent Technologies Ireland, Dublin, Ireland). Only the RNA samples with an RNA integrity number ≥7 were kept for further analysis. Complementary DNA (cDNA) libraries were synthesized with Illumina TruSeq (Illumina, San Diego, CA, USA) according to the manufacturer’s instructions. The sequencing of the cDNA libraries was done in the Hiseq 2000 Illumina platform following the manufacturer’s protocols (Illumina) to generate >60 million paired-end reads (2 × 100 bp; Supplementary Materials Table S2).

### 2.3. RNA-Seq Data Analysis

The quality of the raw RNA sequences was assessed using FastQC v0.11.5 (http://www.bioinformatics.babraham.ac.uk/projects/fastqc/), and the low-quality bases (Phred score <30) were removed with Trimmomatic v0.36 [28] using a sliding window of 4:15, and a minimal read length of 50 bases. The software Bowtie v2.2.9 [29] was used to align the pre-processed RNA sequences to the reference genome *Bos taurus* (Ensemble UMD3.1; Supplementary Materials Table S2) downloaded from the Illumina repository (http://support.illumina.com/sequencing/sequencing_software/igenome.html). The gene count of the reads (Supplementary Materials Table S3) was done with the R packages GenomicFeatures v1.22.13 to build an annotation database from a GTF file and GenomicAlignments v1.6.3 used to manipulate the alignment files (bam files) and produce a gene count matrix [30]. The genes with less than 10 read counts were considered to be lowly expressed and were excluded from further analysis. Raw gene counts were normalized with the trimmed mean of M-values (TMM) normalization in edgeR v3.18.1 [31]. We explored the expression profile in the samples using principal component analysis (PCA) on the logarithm of the number of copies per million (lcpm) with the prcomp function from the R package. The mean-variance relationship of the lcpm was estimated with the voom function [32], and the empirical Bayes moderated t-statistics by the eBayes function [33] was used in the differential gene expression analysis from the package limma v3.32.10 [34]. A linear model was fitted considering the effect of the diets (three levels) and sires (two levels) with the following contrasts: (1) differences between samples taken at 18 months and 30 months (18 vs. 30); (2) Low vs. High MS groups for measurement at 18 mo (18: Low vs. High), and (3) at 30 mo (30: Low vs. High); (4) differences between the age of measurement for the Low MS group (Low: 18 vs. 30); and (5) the High MS group (H: 18 vs. 30). The threshold to define differential expressed genes was set at the FDR adjusted *p*-value <0.05 and the logarithm fold change (log_2_FC) ≥ 1.5. We analyzed the number of shared DE genes between contrasts with the R package VennDiagram v1.6.18 [35].

The R package ClusterProfiler v2.5.5 [36] was used to perform the pathway analysis and gene ontology (GO) analysis using the adjusted *p*-value < 0.05 as a significant threshold. This package uses the latest online version of the Kyoto Encyclopedia of Genes and Genomes (KEGG) and was used to perform an over-representation test to identify the pathways involved in the development of marbling. In the GO analysis, genes were grouped into three categories: biological process (BP), molecular function (MF), and cellular component (CC).

### 2.4. Protein–Protein Interaction Network

To find the most influential group of genes, we performed a protein–protein association network analysis using the differentially expressed genes as input in the public database *STRING* v10.5 (https://string-db.org/) [37]. The genes association scores generated by *STRING* were used in *Cytoscape* v3.5.1 [38] together with the log_2_FC from the contrasts to visualize the gene interaction networks. The genes without association were removed from the network diagram. The number of connections of one gene with others determined the size (degree) of the node, while the thickness of the lines (edges) represented the gene association scores.

## 3. Results

### 3.1. Gene Expression Analysis

In this study, 24 libraries were constructed with 68,198,117 raw reads on average. From these reads, 80% of the cleaned reads were aligned to the reference genome *B. taurus* (UMD3.1). The summary of sequencing quality and mapping is shown in Table 1. The gene counts and a detailed summary of alignment for all libraries are provided in Supplementary Materials Tables S2 and S3, respectively.

**Figure 1:**
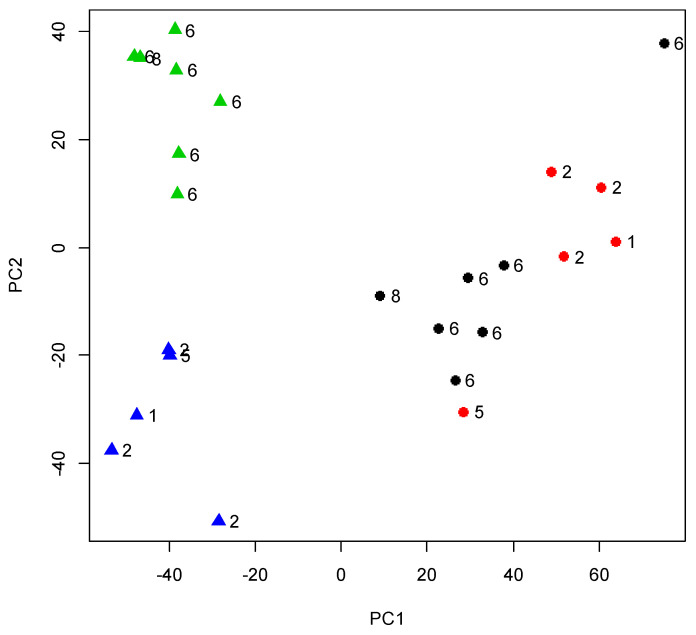
Transcriptomic profile of the Hanwoo muscle at different ages. Principal component analysis (PCA) on log_2_cpm in Hanwoo’s muscle at 18 months (circle) and 30 months (triangle) of age. The numbers indicated the marbling score (recorded at 30 months) while the colors correspond to the MS grouping (High: black and green; Low: red and blue).

After filtering out the genes with low counts, we identified 13,927 expressed genes across the samples. The logarithm of the copies per million (lcpm) was calculated, and the principal component analysis (PCA) showed a clear separation of samples taken at 18 and 30 months in PC1, which explained 39% of the variation in expression Figure 1. On the other hand, PC2 explained 12% of the variation in the expression, splitting the steers with low and high MS into two groups at 30 months of age, however, such a separation was not evident at 18 months.

**Figure 2:**
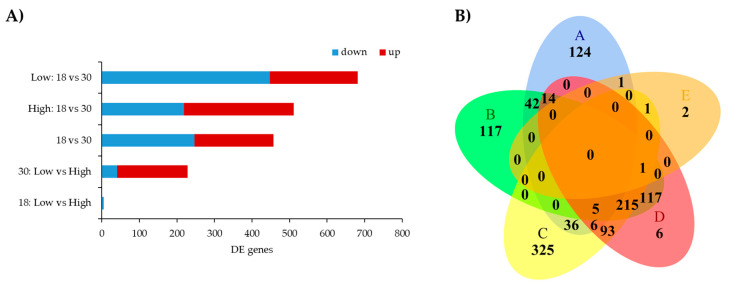
Differentially expressed genes in Hanwoo’s muscle at different ages. (**A**) The number of differentially expressed genes in each contrast (FDR adjusted *p*-value < 0.05 and log_2_FC ≥ 1.5). (**B**) Venn diagram of the number of DE genes shared among the contrasts. (A) 30: Low vs. High; (B) High 18 vs. 30; (C) Low 18 vs. 30; (D) 18 vs. 30; and (E) 18: Low vs. High.

From the five contrasts used to compare transcriptomic differences, in total we found 1883 DE genes (adjusted *p*-value < 0.05 and log_2_FC > 1.5), among them, 782 genes were up-regulated and 1101 down-regulated (Figure 2A). The lowest number of DE genes was five, identified in the contrast 18: Low vs. High, followed by 228 DE genes from the 30: Low vs. High contrast (187 down-regulated and 41 up-regulated genes). More DE genes were found when contrasting expression at different ages, 682 DE genes were the highest number of genes found in the Low: 18 vs. 30 contrast, followed by 511 DE genes found in High: 18 vs. 30 (Figure 2A). The Venn diagram shows the number of differentially expressed genes overlapped between the contrasts. Only five genes were found shared by 30: Low vs. High, Low: 18 vs. 30, High: 18 vs. 30, and 18 vs. 30 contrasts (Figure 2B). From these genes, SCO-spondin (*SSPO*) and the miR-208b (Bta-mir-208b) were up-regulated in High MS, while Midkine (*MDK*), novel gene (ENSBTAG00000003152), and Fibrillin 3 (*FBN3*) were down-regulated in steers with High MS. There were 2, 6, 117, 124, and 326 DE genes uniquely found in 18: Low vs. High, 18 vs. 30, High: 18 vs. 30, 30: Low vs. High, and Low: 18 vs. 30, respectively (Figure 2B). These findings indicate that depending on the marbling group (high or low), there are genes differentially expressed exclusively found between 18 and 30 months.

**Figure 3:**
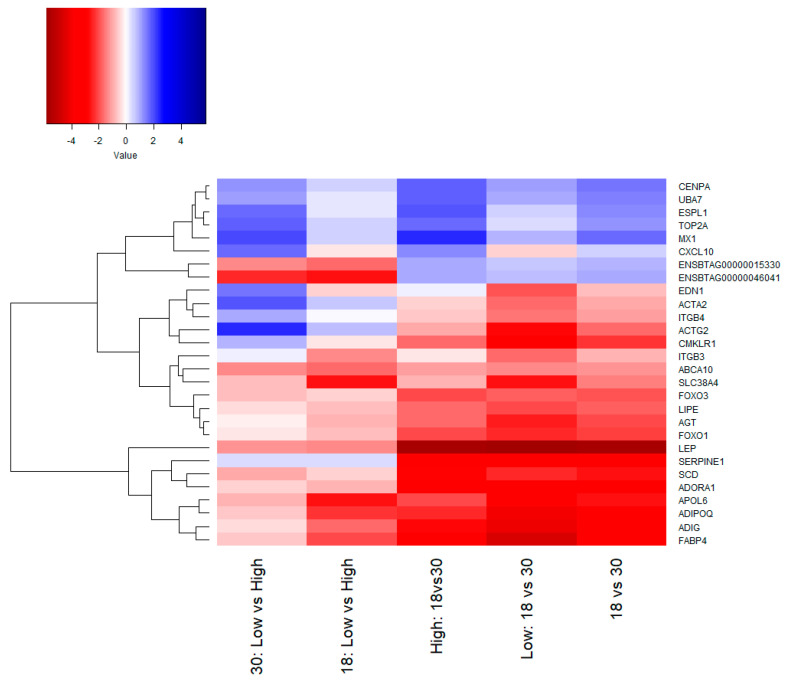
Heatmap comparing the differentially expressed genes. The values shown are the logarithmic fold change (logFC) obtained for each contrast.

In samples at 18 months old, we found five DE genes (Table 2; Figure 3), all of them down-regulated in animals with Low MS: Solute carrier family 38 member 4 (*SLC38A4*), ATP binding cassette subfamily A member 10 (*ABCA10*), Apolipoprotein L6 (*APOL6*), and two novel genes (ENSBTAG00000015330 and ENSBTAG00000046041). The genes *SLC38A4*, *APOL6*, and ENSBTAG00000046041 had the largest difference in expression, however, the FDR adjusted *p*-values barely passed the significance threshold (FDR < 0.05).

### 3.2. Functional Analysis of Differentially Expressed Genes

**Figure 4:**
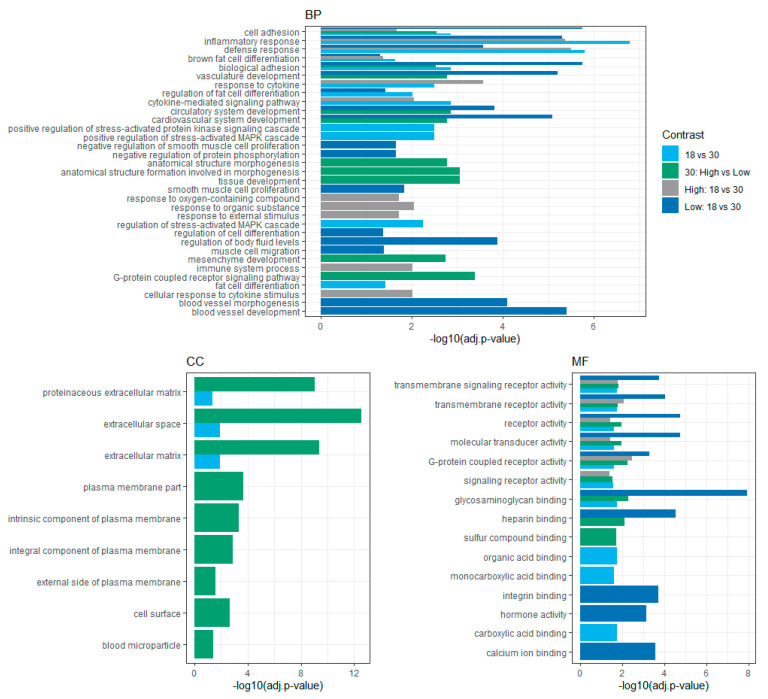
Top 10 significant GO terms and terms related to fat deposition and growth in Hanwoo. BP: biological process; CC: cellular components; MF: molecular function.

The differentially expressed genes were analyzed to determine the enriched terms from the gene ontology (GO) annotation. The top 10 significant GO terms and lipid/growth related terms for the biological process (BP), cellular components (CC), and molecular functions (MF) are shown in Figure 4 for each contrast except 18: Low vs. High, which had only five significant genes.

Some of the most interesting terms in the biological process were regulation of fat cell differentiation, brown fat cell differentiation, fat cell differentiation, inflammatory response, tissue development, and positive regulation of stress-activated MAPK cascade. In the case of the cellular component, extracellular matrix, extracellular space, and proteinaceous extracellular matrix were significant terms. The molecular function category had enrichment of the terms glycosaminoglycan binding, G-protein coupled receptor activity, transmembrane signaling receptor activity, and heparin-binding. The complete table of GO terms can be found in the Supplementary Materials Table S4.

From the gene ontology analysis, the DE genes leptin (*LEP*), mesenteric estrogen-dependent adipogenesis (*MEDAG*), forkhead box O1 (*FOXO1*), adipogenin (*ADIG*), adiponectin (*ADIPOQ*), chemerin chemokine-like receptor 1 (*CMKLR1*), and fatty acid-binding protein 4 (*FABP4*) are involved in the terms fat cell differentiation and brown fat cell differentiation, suggesting a clear effect of their gene expression in final marbling.

The DE genes were also used in the pathway analysis, a summary of the results is shown in Table 3. The most interesting pathways for this trait were related to lipolysis and fat deposition. Results from the contrast in time 18 vs. 30, across the different marbling groups, showed differentially expressed genes involved in the *AMPK* signaling pathway, regulation of lipolysis in adipocytes, and the p53 signaling pathway (Table 3). Interestingly, there was a higher number of DE genes and a larger change in expression (fold change) in the contrast of age for the Low marbling group (Low: 18 vs. 30) compared to this age contrast of the High marbling group. 

### 3.3. Network Analysis

**Figure 5:**
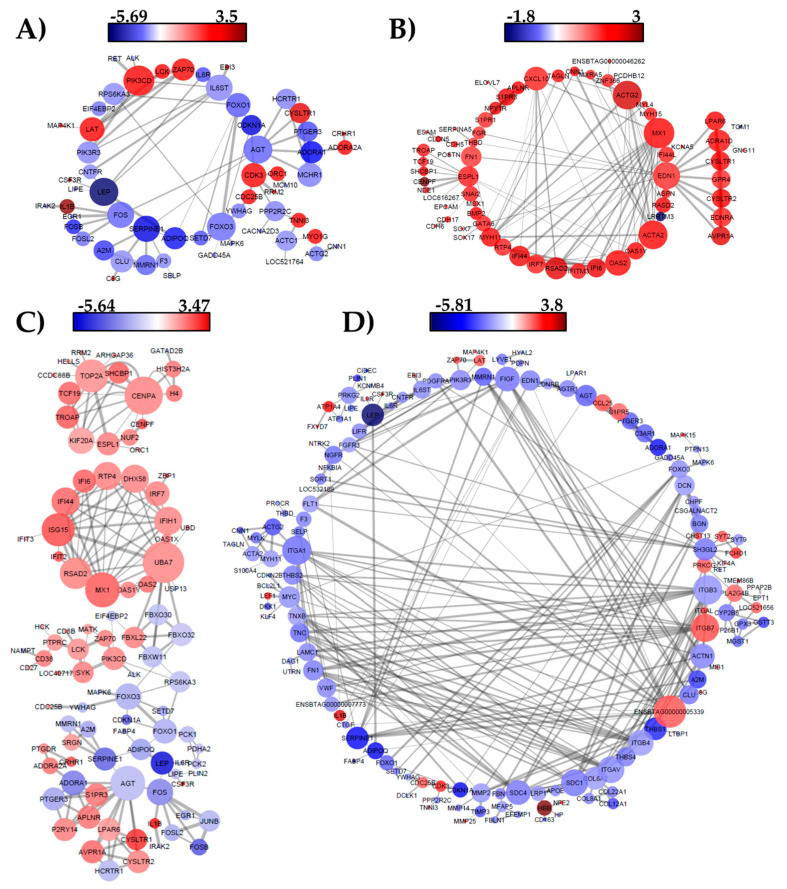
Protein interaction network of DE genes from contrasts. (**A**) 18 vs. 30, (**B**) 30: Low vs. High, (**C**) High: 18 vs. 30, and (**D**) Low: 18 vs. 30. The gray lines are connections (edges) between proteins and the thickness of the lines represents the protein association scores. The circles are proteins in colors according to the logarithmic fold change.

From the protein–protein interaction network analysis, we identified candidate genes based on the number of interactions found. The higher the number of interactions (edges) with other genes, the greater the role of that gene in the activation/repression of other genes and consequently, its effect on a specific phenotype. Genes *AGT*, *SERPINE1*, *ADORA1*, *FOS*, *LEP*, *FOXO1*, and *FOXO3* were involved in the marbling process towards the end of the finishing period (around 30 months) (Figure 5A). These genes were also present at 30 months when contrasting time points for animals in both marbling groups (Figure 5C,D). In addition to the genes already mentioned, at 30 months, the genes *ADIPOQ*, integrin subunit α 1 (*ITGA1*), syndecan 1 (*SDC1*), syndecan 4 (*SDC4*), integrin subunit β 3 (*ITGB3*), and integrin subunit β 4 (*ITGB4*) were up-regulated in animals from the Low marbling group and interacted with many genes (Figure 5D). On the other hand, at 30 months of age, the Low MS phenotype seemed to be associated with the high expression of genes c-x-c motif chemokine ligand 10 (*CXCL10*), actin γ 2 (*ACTG2*), MX dynamin-like GTPase 1 (*MX1*), endothelin 1 (*EDN1*), actin α 2 (*ACTA2*), and extra spindle pole bodies like 1 (*ESPL1*) which interact with many other genes (Figure 5B). This expression pattern in the Low MS group seems to determine a phenotype with a poor deposition of intramuscular fat, and further studies are needed to confirm this hypothesis and determine its primary trigger. The hub gene observed in High marbling steers were *MX1*, ubiquitin-like modifier activating enzyme 7 (*UBA7*), centromere protein A (*CENPA*), and DNA topoisomerase II α (*TOP2A*), whose expression was higher at 18 months (Figure 5C).

## 4. Discussion

### 4.1. Transcriptomic Profile for Marbling Selection at an Early Age

The biggest differences in gene expression were found when comparing different ages in the contrasts (Figure 2A). However, the five genes that were differentially expressed at 18 months between the Low and High marbling group (Table 3) could give an important lead to understand the early development of marbling and to select for the desired marbling phenotype to be expressed at an earlier age. The gene *ABCA10* was one of the promising DE genes that were up-regulated at 18 months in the High marbling group. This gene is a member of the ABCA6-like transporter and was suggested to be involved in lipid homeostasis on human macrophages once the addition of cholesterol suppressed the expression of *ABCA10* in macrophages [39]. There is no report on the function of the *ABCA10* gene in beef cattle, however, the *ABCA* family was involved in the cellular homeostasis of phospholipid and cholesterol in humans [40]. The genes *ABCA6* was previously reported as being involved in intracellular lipid transport processes in human endothelial cells [41], reinforcing the hypothesis of an important role for *ABCA10* in the mobilization and accumulation of intramuscular fat in young Hanwoo steers.

The *SLC38A4* gene expression was more abundant in High MS steers at 18 months, suggesting an early association of this gene with the marbling process. There are no reports in cattle about its gene function, however, one study in perivascular cells from the mice white adipose tissue identified the *SLC38A4* gene as overexpressed in these cells, which activates the expression of *PDGFRα* (a marker for adipocyte precursors) and inhibiting adipocyte differentiation [42]. During embryonic development in mice, *SLC38A4* was identified as being an imprinted gene involved in the regulation of somatic growth. It was observed that mice with deficiencies of *SLC38A4* had reduced body size at birth [43]. In human adipose-derived stromal cells, the gene expression of *SLC38A4* was up-regulated during the adipocyte maturation phase [44].

The higher expression of *APOL6* at 18 months in steers with high marbling phenotype agrees with the study of Menssen et al. [45] where this gene was up-regulated during the adipogenic development of mesenchymal stem cells. The high abundance of *APOL6* and *ABCA10*, as well as their role in lipids transport, suggest that a higher production and movement of fatty acids at early ages in cattle will develop higher marbling scores later in life.

Besides the muscular growth process, the transcriptomic differences found could be to some extent due to changes in the diet because steers were fed with grass while a change to a concentrated diet was after approximately 12 months of age. Nevertheless, in our study, the number of animals in each diet was not large enough to undertake a proper analysis on the influence of the concentrate diet, and the time this feeding started in each marbling group. One possible implication of the unbalanced design is the low power to accurately detect more differentially expressed genes at 18 months. Therefore, the different diet conditions were included in the model to correct for possible confounding effects since some studies had pointed out its effect on gene expression and fat deposition. During the change of diet from forage to concentrate, it was suggested that the synthesis of de novo fatty acids in the adipocytes is not as important as the uptake of fatty acids from circulating triacylglycerides [46]. More recently, Reddy et al. [47] suggested that a high-energy diet during the weaning stage induced the adipogenic gene expression in loin tissue from Hanwoo calves.

### 4.2. Gene Ontology and Pathway Analysis

There are multiple biological processes involved in determining complex traits, for example, in marbling, the score obtained in the meat will directly depend on processes like muscle growth, proliferation, and adipocyte differentiation. To get a global view of the biological function of the DE genes, we performed a functional analysis and identified some enriched terms related to fat differentiation. Moreover, the precise speed and time orchestration in the expression of specific genes seems to determine the final marbling score at 30 months in Hanwoo. In this section, we will discuss only the DE genes with a function relevant to marbling, both those genes found in this study and those that have been described in other studies in cattle or other species.

The expression of different sets of genes is required during specific stages of adipocyte differentiation. In our study, the gene *CMKLR1* was found to be involved in the GO term Regulation of fat cell differentiation. The expression of this gene has been previously reported in subcutaneous adipose tissue during the early stages of differentiation in Japanese Black cattle [48]. In addition, Graugnard et al. [49] suggested that the expression of *FABP4*, glucose-6-phosphate dehydrogenase (*G6PD*), fatty acid synthase (*FASN*), and acetyl-CoA carboxylase α (*ACACA*) is required in the terminal stage of adipocytes differentiation. In Hanwoo, the expression of *FABP4*, stearoyl-CoA desaturase (*SCD*), *PPARG*, and *ADIPOQ*, were significantly higher in high marbling animals [19]

In this study, we identified DE genes that were involved in the AMPK signaling pathway, regulation of lipolysis in adipocytes, p53 signaling pathway, PPAR signaling pathway, PI3K-Akt signaling pathway, and MAPK signaling pathway. The *PPAR* signaling pathway was suggested to play a critical role in bovine adipogenesis [50]. This pathway is activated by fatty acids, and in our study, the *MAPK* signaling pathway was enriched by the genes LOC101906058, *SCD*, perilipin 1 (*PLIN1*), *FABP4*, *ADIPOQ*, phosphoenolpyruvate carboxykinase 2 (*PCK2*), phosphoenolpyruvate carboxykinase 1 (*PCK1*), perilipin 2 (*PLIN2*), carnitine palmitoyltransferase 1A (*CPT1A*), solute carrier family 27 member 5 (*SLC27A5*), and angiopoietin-like 4 (*ANGPTL4*). In the *AMPK* pathway, the AMP-activated protein kinase (AMPK) is a serine/threonine kinase known as a metabolic checkpoint for inhibiting cellular growth. The gene has an important role in controlling mTORC1 and also targets other genes like *p53* and *CDK*, and in fat, AMPK controls lipid metabolism [51]. The *p53* signaling pathway was significant in the 18 vs. 30 and the Low 18 vs. 30 contrasts. In this pathway, the transcriptional activity of *p53* is involved in tumor suppression, cell cycle arrest, apoptosis, and DNA repair. It was previously reported that *p53* is also involved in the negative regulation of MAPK and that the balance between these two pathways will determine the condition of the cell (dead or live) [52]. The gene insulin-like growth factor binding protein 3 (*IGFBP3*), which is also involved in the *p53* signaling pathway, was found to be up-regulated at 30 months in the Low MS group. This gene, together with other insulin-like growth factor signaling genes, were reported to increase their expression during the adipogenic conversion of bovine marrow stromal cells [53].

The *PI3K-Akt* pathway was significantly enriched in our study in the contrasts High: 18 vs. 30 and Low: 18 vs. 30. This pathway is known to control skeletal muscle growth. The *Akt* gene stimulates protein synthesis by activating mTOR and simultaneously regulates autophagy through the transcription factor *FOXO* [54]. In particular, AMPK stimulates *FOXO3* transcriptional activity, which reduces the total protein synthesis in adult muscle [54]. In another study, the expression of *FOXO3* blocked the proliferation of fibroblasts and induced expression of *FOXO1* and *FOXO4* [55]. We found *FOXO1* as a downstream target of important genes for marbling (*FABP4* and *ADIPOQ*). *FOXO1* is important to maintain energy homeostasis, acting as a switch in the synthesis of carbohydrates to lipids in skeletal muscle. The over-expression of *FOXO1* in C2C12 myoblasts increases the fatty acid uptake and increases the CD36 content in the plasma membrane [56]. Moreover, *FOXO1* also plays an important role by repressing the expression of peroxisome proliferator-activator receptor γ (*PPARγ*), a transcription factor that promotes adipogenesis and fat storage. In adipocytes, *FOXO1* interacts with C/EBPα to promote the expression of adiponectin [57]. It was observed in mouse that with the inhibition of *FOXO1*, the PPARγ/RXRα is activated and the cell cycle is suppressed, promoting adipocyte differentiation and maintenance [55].

Together with previous studies, our results emphasize the important function of the *FOXO* genes in muscle tissue to regulate energy homeostasis. Especially in adipose tissue, the induced expression of *FOXO1* ameliorates insulin resistance in the high-fat diet affecting the size of white adipocytes, while in brown adipocytes, it increases oxygen consumption and energy expenditure obesity [58]. Further studies are needed to define the function of *FOXO1* and *FOXO3* in relation to fat deposition in Hanwoo.

In a study on muscle-derived stem cells, the overexpression of *FABP4* promoted the differentiation of genes associated with PPAR signaling and PI3K-Akt signaling pathways [17]. The same pathways were also found in this study (Table 3) for both contrasts High: 18 vs. 30 and Low: 18 vs. 30. Moreover, in the protein network interaction, we observed that *FABP4* is up-regulated at 30 months and has a direct effect on *ADIPOQ* (Figure 5C,D), which coincides with their function in late adipocyte differentiation.

### 4.3. Interaction Network

By analyzing the DE genes at the protein level using an interaction network, we identified the possible networks for the differentially expressed genes at each contrast and recognized 19 genes that seem to have an important role in marbling development. The genes *AGT*, *SERPINE1*, *ADORA1*, *FOS*, *LEP*, *FOXO1*, *FOXO3*, *ADIPOQ*, *ITGA1*, *SDC1*, *SDC4*, *ITGB3*, and *ITGB4* were highly expressed at 30 months in High MS, while the genes *CXCL10*, *ACTG2*, *MX1*, *EDN1*, *ACTA2*, and *ESPL1* were highly expressed at 30 months in Low MS (Figure 3 and Figure 5). Some of these genes have already been signaled by other studies into intramuscular fat or marbling in cattle, however, the function of most of them and their role in bovine adipogenesis have not been explored. This lack of knowledge clearly shows that more studies are needed at the functional level to clarify their role in defining this trait.

The *LEP* gene consists of three exons; its expression occurs mainly in adipocytes and is activated by C/EBPα [59]. There are differences in LEP protein abundance between breeds, with higher expression being reported in Japanese Black, which is considered a breed with a high genetic predisposition for marbling compared to the Holstein breed [60]. Moreover, there is a positive correlation between marbling score and the concentration of protein LEP in serum [61]. The gene *SDC1* was suggested to act as a molecular marker for body size since the polymorphism AA showed superior birth weight and body length in a Chinese breed [62]. The gene *SERPINE1* was up-regulated in Charolais x Red Angus, which developed higher backfat than Hereford x Aberdeen Angus. Additionally, *SERPINE1* was differentially expressed between the high and low backfat groups in Charolais x Red Angus [63].

It has been reported that the use of very extreme phenotypes/genotypes allowed the finding of huge differences in their transcriptome and could provide detailed information of the genes and pathways that determine a trait [24,50]. In our study, we did not use the most extreme phenotypes, but with the subgroups defined by marbling scores, we were able to identify the same genes reported in previous studies and also find other potential candidate genes for marbling development. For example, in the interaction network, we identified the genes *SERPINE1*, *FOS,* and *AGT*, which, together with the genes mentioned above, seem to be determining a High MS phenotype. This finding is supported by the transcriptomic comparison of subcutaneous adipose tissue between Wagyu and Holstein cattle, where Huang et al. [50] suggested that these genes may have a large impact on adipocyte differentiation and adipogenesis. In a time-point study in Wagyu x Hereford, the mRNA expression of *ADIPOQ*, *SCD*, *FAS*, and *THRSP* was highly correlated with intramuscular fat content at 20 and 25 months compared to Piedmontese x Hereford. Wang et al. [24] found that even at an early age (7 months), the expression of *ADIPOQ*, *FABP4*, and *FAS* was markedly elevated in Wagyu x Hereford animals compared to Piedmontese x Hereford. Another interesting gene is *ITGA1,* which was up-regulated in animals from the Low marbling group. The knockdown of the gene *ITGA1* in mice promoted lipid accumulation and facilitated hepatic insulin action [64]. In cattle, this gene was found to be significantly up-regulated in intramuscular fat [65].

In our study, the up-regulated expression of the *MX1* gene and its connections with other genes in the interaction network in the Low MS group at 30 months suggest that this gene is involved in the Low marbling phenotype. However, the *MX1* gene is also up-regulated in the High MS group, but at 18 months, which seems to be higher when compared with the Low MS group (Figure 4). The role of this gene needs to be investigated in further studies. Based on previous genomic studies, the B recessive allele of *MX1* showed an association with carcass traits and meat quality, reflected in a higher allele frequency of this allele for a Chinese local pig breed, which also has been described as having better meat quality than the Landrace pig [66]. Furthermore, Iqbal et al. [67] identified a significant SNP close to *MX1* and suggested this gene as a candidate for backfat thickness in pigs. *EDN1* gene is a vasoconstrictor and mitogen with a role in hypertrophy and heart failure. It was reported that treatment with exogenous myostatin in C2C12 myoblast induced the expression of *EDN1,* and it was followed by a reduction of MAPK signaling cascade and G-protein related signaling genes [68].

## 5. Conclusions

In conclusion, among the 1883 differentially expressed genes, we identified five new potential early age markers for marbling development in Hanwoo cattle (*SLC38A4*, ENSBTAG00000015330, ENSBTAG00000046041, *ABCA10*, and *APOL6*). Moreover, we described the transcriptomic differences between steers with Low and High marbling scores and reported some DE genes that have not yet been described as being involved in marbling. Finally, we recognized the important role of *FOXO1* for its implication in multiple pathways (cellular growth, cell cycle arrest, apoptosis, energy homeostasis) and its interaction with important genes for fat deposition (as *FABP4* and *ADIPOQ*). However, further functional studies on these genes are needed, and more samples with adipocyte differentiation in Hanwoo cattle should be analyzed to better understand their role in fat development or accumulation and the possible use of these genes to predict marbling in cattle at an earlier age.

## Figures and Tables

**Table 1:** Summary of sequencing reads aligned for low and high marbling (MS) at 18 and 30 months.

	18 Months		30 Months	
	High MS	Low MS	High MS	Low MS
Total reads	66,334,797	68,966,322	67,403,192	70,088,156
Pair reads mapped	32,628,467	33,654,496	33,488,986	33,644,083
Unique matched	15,865,630	16,234,604	15,904,205	16,064,734
Multimatched reads	3,722,653	4,588,413	4,211,752	4,346,990
Overall map	80.8%	80.6%	79.9%	80.3%

**Table 2:** Differentially expressed genes in Hanwoo steers at 18 months old.

Gene	logFC	Average Expression	*p*-Value	*FDR*
***ABCA10***	−1.64	2.94	1.97 × 10^−5^	0.046
**ENSBTAG00000015330**	−1.68	1.43	1.75 × 10^−5^	0.046
**ENSBTAG00000046041**	−2.57	−0.17	1.79 × 10^−5^	0.046
***APOL6***	−2.59	−1.08	2.51 × 10^−5^	0.049
***SLC38A4***	−2.62	0.14	1.06 × 10^−5^	0.046

**Table 3:** Enrichment analysis of the pathways related to fat deposition and growth in Hanwoo. Statistical significance represented by the adjusted *p*-value: * *p* <0.05; ** *p* < 0.01; ***p < 0.001. Symbols indicate unique genes in 18 vs. 30 (*), High: 18 vs. 30 (+), and Low: 18 vs. 30 (#) contrast.

Pathway	18 vs. 30	High 18 vs. 30	Low 18 vs. 30	Genes
AMPK signaling pathway (bta04152)	12 **	12 *	13 **	*LIPE*, *FOXO3*, *LEP*, *LOC101906058*, *SCD*, *FOXO1*, *PPP2R2C*, *PFKFB3*, *ADIPOQ*, *PCK2*, PCK1*, PIK3CD*, *PIK3R3*+, *PIK3CD*+, *MLYCD*#, *CPT1A*#, *PIK3R3*#
Regulation of lipolysis in adipocytes (bta04923)	7 *		7 *	*LIPE*, *ADORA1*, *PIK3R3*, *PLIN1*, *FABP4*, *PTGER3*, PIK3CD*, *PRKG2*#
p53 signaling pathway (bta04115)	7 *		9 *	*CDKN1A*, *THBS1*, *SERPINE1*, *SESN3*, *GADD45A*, *TP73*, RRM2*, *IGFBP3*#, *ZMAT3*#, *PERP*#
PPAR signaling pathway (bta03320)		8 *	9 *	*LOC101906058*, *SCD*, *PLIN1*, *FABP4*, *ADIPOQ*, *PCK2*, *PCK1*+, *PLIN2*+, *CPT1A*#, *SLC27A5*#, *ANGPTL4*#
PI3K-Akt signaling pathway (bta04151)		3 *	32 ***	*FOXO3*, *FOXO1*+, *PIK3CD*+, *THBS4*#, *CDKN1A*#, *BCL2L1*#, *COL6A2*#, *VWF*#, *LAMC1*#, *PDGFRA*#, *THBS1*#, *ITGB4*#, *ITGAV*#, *VEGFA*#, *FLT1*#, *IL6R*#, *ITGA1*#, *TNXB*#, *LPAR1*#, *FN1*#, *YWHAG*#, *VEGFD*#, *TNC*#, *ITGB3*#, *PIK3R3*#, *THBS2*#, *MYC*#, *RELN*#, *NGFR*#, *PPP2R2C*#, *PCK2*#, *ITGB7*#, *FGFR3*#, *CSF3R*#
MAPK signaling pathway (bta04010)			7 *	*PDGFRA*, *MAP3K2*, *IL1B*, *CD14*, *PLA2G4B*, *NTRK2*, *TAOK1*, *ELK4*, *HSPA1A*, *MYC*, *PRKCG*, *GADD45A*, *RASGRP2*, *FGFR3*, *MAP4K1*, *CDC25B*, *HSPA6*

## Supplementary Materials

The following are available online at https://www.mdpi.com/2073-4425/11/11/1381/s1, Table S1: Composition of the concentrate diet; Table S2: Alignment of the reads using *Bos taurus* (UMD3.1) reference genome from Ensembl. The Excel file containing the summary of paired reads were aligned to the reference. The reads that aligned discordantly once (AD) and the reads that aligned concordantly (AC) zero times, once, and more than once are presented; Table S3: Gene expression counts. Text file with the counts for each gene and samples; Table S4: Gene ontology terms from all the contrasts. Excel file containing the terms cellular component, biological process, and molecular function for each differentially expressed gene from the contrasts.
