# Supplementary material for: Differential Gene Expression in Longissimus Dorsi Muscle of Hanwoo Steers—New Insight in Genes Involved in Marbling Development at Younger Ages
Source: Genes (Basel). 2020 Nov 21;11(11):1381. doi: 10.3390/genes11111381 (PMC7700136; doi:10.3390/genes11111381)
Supplement: Supplementary file 1 [file genes-11-01381-s001.zip › Table S1. Composition of the concentrate diet..docx]

**Table S1.** Composition of the concentrate diet.

| **Ingredients** | **Concentrate (%)** |
| --- | --- |
| Corn | 40.0 |
| Lupin seed | 1.0 |
| Wheat | 10.0 |
| Wheat bran | 10.0 |
| Gluten feed | 18.0 |
| Oats bran | 2.0 |
| Palm oil meal | 10.0 |
| Sodium bicarbonate | 2.0 |
| Salt | 0.5 |
| Molasses | 5.0 |
| Vitamin and mineral premix | 1.5 |
| Total | 100 |
| Nutritional characteristics of the diet | (%) |
| Dry matter | 87.9 |
| Crude protein | 12.4 |
| Ether extract | 3.7 |
| Crude ash | 6.1 |
| Neutral detergent fiber | 21.0 |
| Acid detergent fiber | 9.9 |
